# Association of Cigarette Smoking With Male Cognitive Impairment and Metal Ions in Cerebrospinal Fluid

**DOI:** 10.3389/fpsyt.2021.738358

**Published:** 2021-11-19

**Authors:** Hui Li, Qingshuang Mu, Yimin Kang, Xiaoyu Yang, Ligang Shan, Meiling Wang, Cunbao Li, Yanlong Liu, Fan Wang

**Affiliations:** ^1^Department of Biomedical Engineering, College of Engineering, Peking University, Beijing, China; ^2^Xinjiang Key Laboratory of Neurological Disorder Research, The Second Affiliated Hospital of Xinjiang Medical University, Urumqi, China; ^3^Key Laboratory of Psychosomatic Medicine, Inner Mongolia Medical University, Hohhot, China; ^4^Beijing Jishuitan Hospital, Beijing, China; ^5^Department of Anesthesiology, The Second Affiliated Hospital of Xiamen Medical College, Xiamen, China; ^6^School of Mental Health, Wenzhou Medical University, Wenzhou, China; ^7^The Affiliated Kangning Hospital, Wenzhou Medical University, Wenzhou, China; ^8^Beijing Hui-Long-Guan Hospital, Peking University, Beijing, China

**Keywords:** cigarette smoking, cognitive impairment, metal ion, cerebrospinal fluid, male

## Abstract

**Objective:** Cigarette smoking might accelerate cognitive impairment; however, this has never been investigated using human cerebrospinal fluid (CSF). We conducted this study to investigate the association between cigarette smoking and cognitive impairment through metal ions in CSF.

**Methods:** We obtained 5-ml CSF samples from routine lumbar puncture procedures in patients undergoing anterior cruciate ligament reconstruction before surgery in China. A total of 180 Chinese males were recruited (80 active smokers and 100 non-smokers). We measured specific cigarette-related neurotoxic metal ions in CSF, including iron, copper, zinc, lead, aluminum, and manganese. Sociodemographic data and history of smoking were obtained. The Montreal Cognitive Assessment (MoCA) was applied.

**Results:** Active smokers had fewer years of education (11.83 ± 3.13 vs. 13.17 ± 2.60, *p* = 0.01), and higher age (33.70 ± 10.20 vs. 29.76 ± 9.58, *p* = 0.01) and body mass index (25.84 ± 3.52 vs. 24.98 ± 4.06, *p* =0.03) than non-smokers. Compared to non-smokers, active smokers had significantly higher CSF levels of iron, zinc, lead, and aluminum and lower MoCA scores (all *p* < 0.05). Average daily numbers of cigarettes smoked negatively correlated with the MoCA scores (*r* = −0.244, *p* = 0.048). In young smokers, CSF manganese levels negatively correlated with MoCA scores (*r* = −0.373, *p* = 0.009).

**Conclusions and Relevance:** Cigarette smoking might be associated with male cognitive impairment, as shown by lower MoCA scores and higher levels of CSF iron, zinc, lead, and aluminum in active smokers. This might be early evidence of cigarette smoking accelerating male cognitive impairment.

## Introduction

Evidence suggests that cigarette smoking might accelerate brain aging (1). Metals found in cigarette smoke have been known to accumulate in tissues and fluids (2, 3), such as iron, copper, zinc aluminum, manganese and lead (4, 5). Metal accumulation in the nervous system could lead to heavy metal toxicity and accelerate cognitive impairment (6–8). Neurodegeneration, characterized by cognitive impairment, is the most common manifestation of heavy metal toxicity (9).

Increasing evidence suggests that dysregulation of iron, copper, and zinc homeostasis contributes to several neurodegenerative diseases (10, 11). Iron is involved in many fundamental biological processes in the brain, including oxygen transport, DNA synthesis, and mitochondrial respiration. Iron accumulation might be an essential factor contributing to neurodegenerative processes such as Alzheimer's disease (AD) (12). Copper is an active oxidation-reduction metal, as is iron, and both share toxicological consequences (13). Elevated copper levels may result in the generation of reactive oxygen species (ROS), DNA damage, and mitochondrial dysfunction (14). Disruption of the tightly regulated copper homeostasis in the brain can result in severe neurological malfunction and neurodegeneration. Studies suggested that the pathogenesis of neurodegenerative disorders such as AD involves an imbalanced copper homeostasis in the brain (15–17). Zinc's primary role is to stabilize the structure of several proteins, including signaling enzymes at all levels of cellular signal transduction and transcription factors. Excess zinc levels promote ROS production in the mitochondria, disrupting activities of metabolic enzymes, and activating apoptotic processes (18). Disruption of zinc homeostasis has been associated with AD (19).

Manganese is essential for human development and brain function. Excessive manganese levels are neurotoxic, as they disrupt mitochondrial function and induce oxidative stress (20). Chronic manganese exposure produces a cellular stress response that leads to neurodegenerative changes (21, 22). Mass spectrometry studies demonstrated that aluminum crosses the blood-brain barrier and accumulates in a semipermanent manner (23). Oral administration of aluminum to AD mice induced an increase in the amount of amyloid beta-protein and its deposition in plaques and aluminum to induce neurofibrillary degeneration and promote the appearance of tangle-like structures resembling AD patients (24). Also, aluminum exhibits an affinity for phosphates, thereby making DNA, RNA, and ATP perfect targets, affecting gene expression (6). A basic cellular and animal study elucidated the toxic actions of lead within the central nervous system (25). Lead has also been shown to induce latent changes in the aging brain and has been implicated in the pathogenesis of neurodegenerative diseases, particularly AD (26). Lead exposure in childhood could increase neurodegenerative disease risk in adulthood (25).

Components of cigarette smoke rapidly enter the brain in several ways and may cause the accumulation of these metal ions. Metal toxicity is associated with several neurodegenerative diseases, depending on levels of metal ions in the brain (27, 28). Nevertheless, the role of those metal ions in the association between cigarette smoking and cognitive impairment has never been reported. Therefore, this study was conducted to investigate the association of cognition and metal ions levels in CSF of cigarette smokers to further explore and support the effects of cigarette smoking on cognitive impairment.

## Materials and Methods

### Participants

Because there are few female subjects smokers in China, 180 Chinese males scheduled for anterior cruciate ligament reconstruction surgery were recruited from September 2014 to January 2016 [method as described in the literature (29)]. Of these, 80 were active smokers, and 100 were non-smokers. Sociodemographic data, including age, years of education, and body mass index (BMI), were collected. Clinical data, including a history of substance abuse and dependence, were obtained according to self-report and confirmed by the next of kin and family members. Exclusion criteria were as follows: (1) a family history of psychosis or neurological diseases, or CNS diseases determined by the Mini-International Neuropsychiatric Interview; and (2) systemic diseases based on the medical history and admitting diagnosis.

Participants who had never smoked and had no history of substance abuse or dependence were assigned to the non-smoker group. Active smokers were defined as those who consumed half a pack of cigarettes (half pack = 10 cigarettes) or more per day for more than 1 year. Smokers who smoked fewer than 10 cigarettes per day were excluded. No participants had a history of alcohol abuse or any psychiatric disorders, according to the Diagnostic and Statistical Manual of Mental Disorders, 4th Edition. All subjects were all independent without kinship. Active smokers were further grouped into younger smokers (*n* = 59, <40 y/o) and elder smokers (*n* = 21, ≥40 y/o), according to the literature (30). Based on World Health Organization criteria, active smokers were divided into moderate smokers (*n* = 46, >10 and <20 cigarettes per day for more than 1 year) and heavy smokers (*n* = 34, ≥ 20 cigarettes or more per day for more than 1 year. The maximum in this study was 40 cigarettes per day).

The present study was approved by the Institutional Review Board of Inner Mongolian Medical University and performed in accordance with the Declaration of Helsinki. Written informed consent was obtained from all subjects.

### Assessments, Biological Sample Collection, and Laboratory Tests

Smoking-related habit variables were obtained from active smokers, including age at smoking onset, years of cigarette smoking, average daily amount of cigarette smoking, and maximum daily amount of cigarette smoking. Cognition was assessed using the Montreal Cognitive Assessment (MoCA), a brief cognitive screening tool with high sensitivity and specificity for mild cognitive impairment (MCI), using a cutoff score of 26, with those scoring 25 or below being suspected of having MCI (31). The MoCA is a tool to differentiate healthy cognitive aging from MCI (32).

We recorded levels of high-density lipoprotein (HDL), low-density lipoprotein (LDL), alanine aminotransferase test (ALT), cholesterol (CHO), triglyceride (TG), gamma-glutamyl transferase (GGT) and aspartate aminotransferase (AST) which came from routine tests of the subjects to evaluate physical condition on admission. These peripheral metabolic marker levels were measured in the morning on the second hospital day after an overnight fasting period using a biochemistry analyzer (HITACH 7600, Hitachi Co., Tokyo, Japan).

Lumbar puncture is part of standard clinical procedure for patients undergoing anterior cruciate ligament reconstructive surgery in China. A licensed anesthetist performed a lumbar puncture in the morning before surgery, and a 5-ml CSF sample was obtained via intrathecal collection followed by immediately frozen at −80°C for storage. It takes <1 h to complete the entire anterior cruciate ligament reconstruction operation. The time from hospitalization to surgery was a maximum of 2 days.

Analyses were performed to measure CSF levels of iron, copper, zinc, lead, aluminum and manganese by atomic absorption spectrophotometry (33). Laboratory technicians were blinded to clinical data.

### Statistical Analysis

The normality of all variables was assessed using the Shapiro–Wilk test. Only the distribution of zinc, cooper, systolic pressure, and diastolic pressure were normally distributed (all *p* > 0.05). Consequently, the Mann-Whitney rank sum test was used (Table 1). The normality of the residuals from these models was assessed using the Shapiro–Wilk test. The homoscedasticity of residuals of the variances was verified using Levene's test; the residuals were all equally distributed (*p* > 0.05), except iron (*p* = 0.007). Therefore, analysis of covariance (ANCOVA) was used to compare differences in raw biomarkers between groups (Tables 2, 3). Multi-collinearity among covariates was estimated using tolerance and the variance inflation factor (VIF), using cutoffs thresholds for tolerance of < 0.1 and VIF >10 (34). Partial correlation analysis was performed to test the correlation between smoking habit variables and MoCA and between smoking habit variables and metal levels.

**Table 1:** The differences of clinical characteristics between non-smokers and active smokers.

**Variables**	**Non-smokers (***n*** = 100) (Mean ± SD) (Median, IQR)**	**Active smokers (***n*** = 80) (Mean ± SD) (Median, IQR)**	***p***
Age (years)	29.76 ± 9.58 (27.50, 14.75)	33.7 ± 10.20 (31.50, 15.25)	0.01*
Education (years)	13.17 ± 2.60 (15.00, 4.00)	11.83 ± 3.13 (11.00, 7.00)	0.01*
BMI (Kg/m^2^)	24.98 ± 4.06 (24.22, 4.73)	25.84 ± 3.52 (26.18, 4.91)	0.03*
Systolic pressure (mmHg)	129.88 ± 12.87 (130, 16.75)	127.81 ± 13.87 (128.50, 19.25)	0.34
Diastolic pressure (mmHg)	75.12 ± 9.52 (76.5, 29)	76.31 ± 11.57 (77.00, 14.75)	0.51
HDL (mmol/L)	1.28 ± 0.33 (1.25, 0.30)	1.23 ± 0.28 (1.18, 0.27)	0.44
LDL (mmol/L)	2.66 ± 0.73 (2.66, 0.83)	2.65 ± 0.64 (2.63, 0.71)	0.88
ALT (U/L)	30.47 ± 23.08 (25.50, 9.00)	31.95 ± 22.99 (28.00, 15.75)	0.62
CHO (mmol/L)	4.72 ± 0.95 (4.69, 0.99)	4.79 ± 0.85 (4.75, 1.00)	0.60
TG (mmol/L)	1.85 ± 1.09 (1.61, 1.13)	1.76 ± 1.21 (1.50, 0.86)	0.17
GGT (U/L)	40.90 ± 31.26 (30.00, 26.75)	48.59 ± 47.07 (30.50, 35.50)	0.38
AST (U/L)	21.59 ± 9.49 (20.00, 8.00)	20.59 ± 7.38 (20.00, 9.75)	0.71
MoCA	26.77 ± 1.91 (27.00, 2.00)	25.70 ± 2.39 (26.00, 2.75)	0.003*
CSF iron (μmol/L)	11.24 ± 1.72 (11.15, 2.53)	14.59 ± 1.22 (14.60, 1.80)	<0.001*
CSF copper (mg/L)	0.68 ± 0.09 (0.67, 0.12)	0.68 ± 0.07 (0.68, 0.11)	0.91
CSF zinc (μmol/L)	11.19 ± 1.33 (11.04, 2.13)	12.14 ± 1.58 (12.96, 2.26)	<0.001*
CSF lead (μg/L)	120.05 ± 13.04 (121.64, 20.38)	139.13 ± 12.02 (137.23, 15.97)	<0.001*
CSF aluminum (μmol/L)	0.88 ± 0.09 (0.90, 0.12)	0.93 ± 0.09 (0.94, 0.11)	<0.001*
CSF manganese (nmol/L)	0.0307 ± 0.0061 (0.033, 0.009)	0.0288 ± 0.0059 (0.029, 0.010)	0.038*

*HDL, high-density lipoprotein; LDL, low-density lipoprotein; ALT, alanine aminotransferase; CHO, cholesterol; TG, triglyceride; GGT, gamma-glutamyl transferase; AST, aspartate aminotransferase; MoCA, Montreal Cognitive Assessment; CSF, cerebral spinal fluid; SD, standard deviation; BMI, body mass index; IQR, Interquartile range*.

*All data were reported as mean ± SD using Mann-Whitney sum tests and median with IQR*.

* *p < 0.05*.

**Table 2:** The differences of MoCA and metal levels between non-smokers and active smokers.

**Object**	**Mean differences**	**95% CI**	** $\eta_{p}^{2}$ **	***F***	***p***
MoCA	0.656	0.010, 1.302	0.023	4.02	0.047*
CSF Iron (μmol/L)	−3.089	−3.740, −2.437	0.350	87.61	<0.001*
CSF Copper (mg/L)	−0.036	−0.086, 0.014	0.012	2.00	0.159
CSF Zinc (μmol/L)	−1.604	−2.252, −0.856	0.100	17.92	<0.001*
CSF Lead (μg/L)	−11.436	−18.059, −4.813	0.067	11.63	0.001*
CSF Aluminum (μmol/L)	−0.054	−0.102, −0.005	0.029	4.83	0.029*
CSF Manganese (nmol/L)	0.000	−0.003, 0.004	0.000	0.03	0.859

*ANCOVA, analysis of covariance; CI, confidence interval; $\eta_{p}^{2}$, partial eta squared values; MoCA, Montreal Cognitive Assessment; BMI, body mass index*.

*ANCOVA was used to calculate the differences between two groups; Age and education were treated as covariates of the differences of MoCA scores. The differences of the metal's levels were calculated with age, BMI, education, and other metals as covariates; Mean differences is the mean of non-smokers minus the mean of active smokers*.

* *p < 0.05*.

**Table 3:** The differences of CSF biomarker levels between subgroups in active smokers.

**Objects**	**Younger/elder smokers (*****n*** **= 59/21)**			**Moderate/heavy smokers (*****n*** **= 46/34)**		
	**Mean differences**	**95%CI**	***p***	**Mean differences**	**95%CI**	***p***
MoCA	−0.037	−2.418, 2.343	0.975	1.605	−0.147, 3.357	0.072
CSF Iron (μmol/L)	0.606	−0.717, 1.928	0.364	0.701	−0.268, 1.671	0.153
CSF Copper (mg/L)	−0.009	−0.092, 0.073	0.820	0.004	−0.058, 0.065	0.905
CSF Zinc (μmol/L)	−0.352	−2.123, 1.420	0.693	0.210	−1.116, 1.536	0.753
CSF Lead (μg/L)	−2.854	−15.916, 10.208	0.664	−5.610	−15.197, 3.976	0.247
CSF Aluminum (μmol/L)	−0.069	−0.169, 0.030	0.167	−0.038	−0.113, 0.038	0.319
CSF Manganese (nmol/L)	−0.005	−0.012, 0.001	0.114	−0.002	−0.007, 0.003	0.386

*CSF, cerebral spinal fluid; MoCA, Montreal Cognitive Assessment; ANCOVA, analysis of covariance; CI, confidence interval*.

*ANCOVA was used to calculate the differences between two groups; Age, education, metals, and smoking habits were treated as covariates of the differences of MoCA scores. The differences of metals levels were calculated with BMI, education, metals, and smoking habits as covariates; Mean differences is the mean of younger/moderate minus the mean of elder/heavy smokers, respectively*.

All statistical analyses were performed using IBM SPSS Statistics for Windows, Version 22.0 (IBM Corp., Armonk, NY, USA). Figures were created using GraphPad Prism version 8 (GraphPad Software Inc.). Since the variables under study might be heavily inter-dependent, especially the metals, and a possible effect worthy of further study did not wish to be missed in an exploratory context (35), therefore the Bonferroni correction was not conducted in the present study. All tests were two-sided, and the significance threshold was set at *p* < 0.05.

## Results

### Demographic and Clinical Characteristics

Compared to active smokers, non-smokers had significantly more years of education (13.17 ± 2.60 years vs. 11.83 ± 3.13 years, *p* = 0.01) and lower BMI (24.98 ± 4.06 vs. 25.84 ± 3.52 kg/m^2^, *p* = 0.03), and were younger (29.76 ± 9.58 years vs. 33.7 ± 10.20 years, *p* = 0.01). There were no differences between groups for other sociodemographic and clinical characteristics (Table 1). There were no significant correlations between BMI and metal levels and between age and metal levels in each group (all *p* > 0.05, Supplementary Material 1).

### Cognition and CSF Metals

**Figure 1:**
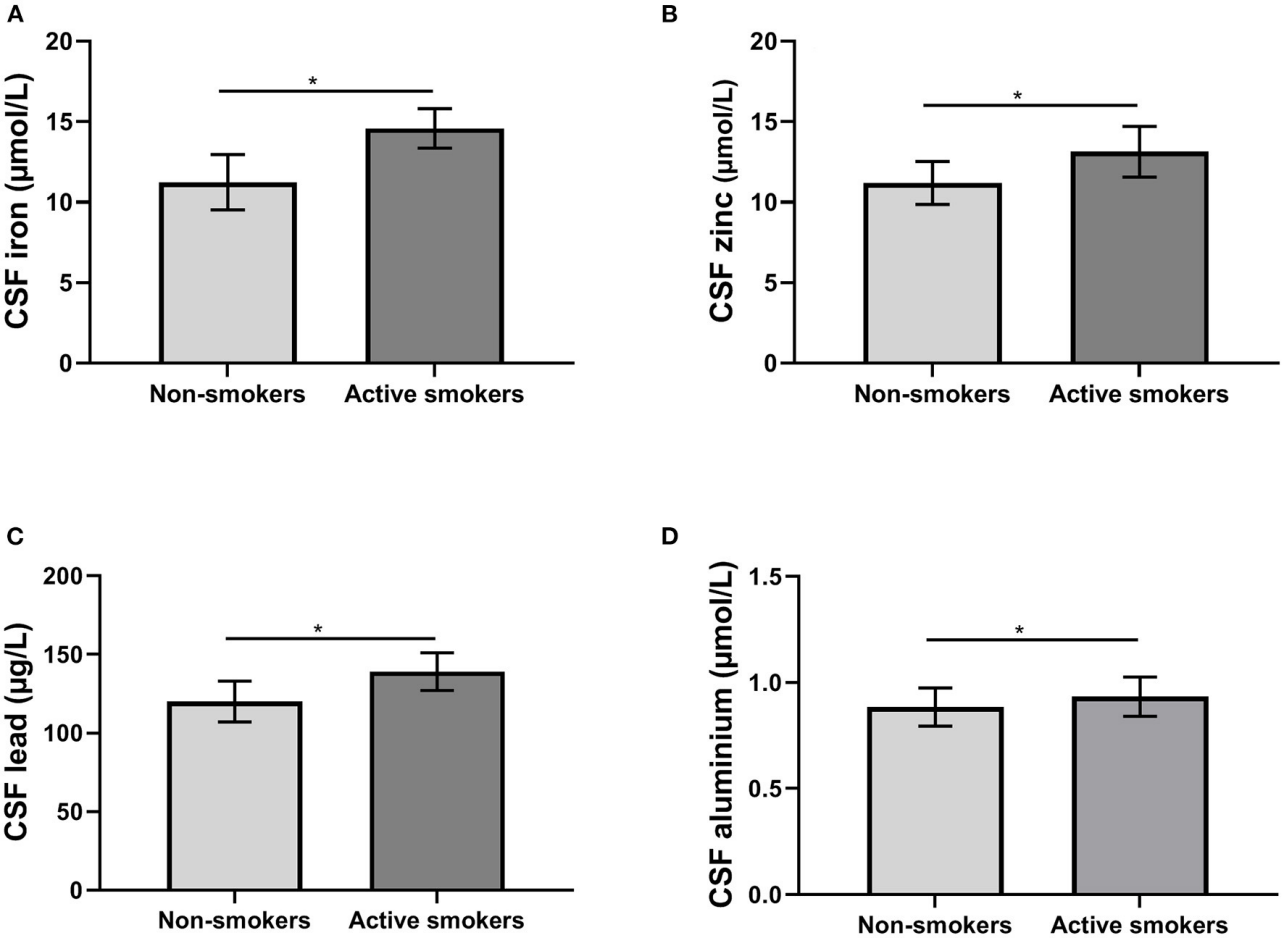
The differences of biomarkers in cerebral spinal fluid (CSF) between groups. **(A)** The differences of iron levels; **(B)** the differences of zinc levels; **(C)** the differences of lead levels; **(D)** the differences of aluminum levels. *p < 0.05.

Using ANCOVA with age and education of years as covariates, the MoCA scores of non-smokers were significantly higher. Using ANCOVA with age and education as covariates, CSF levels of iron, zinc, lead, and aluminum were significantly higher in active smokers (all *p* < 0.05) (Table 2; Figure 1). No correlation was found between metal ions, age and MoCA scores in either group.

### Correlation and Difference in Active Smokers

Stepwise multiple regression analyses of six metals showed that no variable was removed from models (all tolerance >0.5 and VIF <3). Age was removed from models in active smokers (Table 3) since collinearity of age with age at smoking onset and years of cigarette smoking (both tolerance < 0.1 and VIF >30).

We calculated the correlation between smoking habit variables and MoCA, and smoking habit variables and the metals. Considering collinearity of age with age at smoking onset and years of cigarette smoking (both tolerance < 0.1 and VIF >30), average daily amount of cigarette smoking was negatively correlated with MoCA scores (*r* = −0.244, *p* = 0.048) with years of education and other smoking habits as covariates. With BMI, years of education, smoking habits, and other metals as covariates, there were no correlations between smoking habits and metal levels (all *p* > 0.05).

Active smokers were grouped into younger smokers (*n* = 59) and elder smokers (*n* = 21); 46 were moderate smokers, and 34 were heavy smokers. No differences were observed in CSF metal levels between younger and elder smokers or between moderate and heavy smokers (*p* > 0.05) with BMI, education, metals, and smoking habits as covariates. Compared to heavy smokers, there was a trend of higher MoCA scores in moderate smokers (*p* = 0.072) adjusted for age, years of education, metals, and smoking habits.

**Figure 2:**
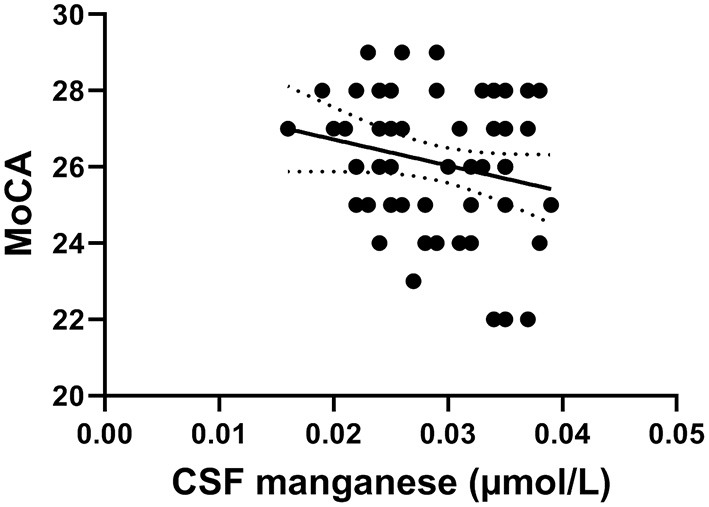
The negative correlation of manganese levels with MoCA scores in young smokers (*r* = −0.349, *p* = 0.012).

Figure 2 shows that CSF manganese levels negatively correlated with MoCA scores in young smokers (*r* = −0.373, *p* = 0.009) adjusted for years of education, BMI, other metals, and smoking habits.

## Discussion

This is the first study to investigate the association of cigarette smoking and cognitive impairment through metal ions of CSF. We found that cigarette smoking might be associated with cognitive impairment, as shown by higher levels of iron, zinc, lead, and aluminum in CSF and lower MoCA scores in active smokers than non-smokers. The average daily amount of cigarette smoking was negative correlation with the MoCA scores.

Cigarette smoke might accelerate aging and cognitive impairment, including enhancing the risk of AD (36). Compared to non-smokers, middle-aged male smokers experienced a faster cognitive decline in global cognition and executive function (37). Heavy smoking is associated with cognitive impairment and cognitive decline in middle age (38). The differences in metal ion levels also might suggest an association between cigarette smoking and cognitive impairment.

Cigarette smoke can affect iron transporters (39); the principal ingredient (nicotine) blocks iron uptake by inhibiting iron release from transferrin and endocytosis (40). Brain iron is abnormally elevated early in several neurodegenerative disorders that impact memory, including AD (41). Studies reported a role for iron in neurodegenerative disorders, including increased iron levels in AD brains and iron involvement in the process of aging (42, 43). Moreover, iron is considered to accelerate cognitive impairment by inducing oxidative stress, ferroptotic cell death, or inflammatory responses (44). A recent cross-sectional study found a negative impact of chronic tobacco smoking on adult neuropsychological function, including alternating attention, working memory, short-term memory, long-term memory, processing accuracy, and executive function (45). In the present study, consistent with these previous studies, higher CSF iron levels and lower MoCA scores in active smokers, and average daily amount of cigarette smoking negative correlation with MoCA scores showed that cigarette smoking promoted the kind of change, suggesting cigarette smoking accelerating cognitive impairment.

Metallothionein (MT) is a group of metal-binding proteins in the blood-brain barrier. MT regulates the intracellular homeostasis of zinc. Because the copper-MT binding constant is much larger relative to zinc, MT exchanges zinc for copper when excess copper is present to defend against the more toxic copper (46). This phenomenon could explain the lack of difference in CSF copper levels between our two groups. A study showed that serum copper and zinc concentrations were significantly higher in smokers than in non-smokers; however, in rats, copper-zinc ratios in the liver, kidney, lung, and brain were significantly altered by nicotine treatment (47). This finding could partly explain higher CSF zinc levels in active smokers in our study. Zinc homeostasis is altered in aging, and there is deranged brain zinc homeostasis in AD. Although there are controversial views regarding zinc supplementation preventing AD pathology (48, 49), excessive zinc intake can lead to degeneration of cognitive function (50–52). This finding further suggests that higher CSF zinc levels as a feature of cigarette smoking accelerating cognitive impairment. Both iron and zinc have a higher binding affinity to Aβ and can promote its aggregation. Increased neuronal iron and zinc also bind to tau protein and facilitate the formation of neurofibrillary tangles to accelerate cognitive decline (6). Therefore, in conjunction with lower MoCA scores, levels of both metal ions were higher in CSF of active smokers, strongly suggesting the effects of cigarette smoking on cognitive impairment.

Manganese is an essential metal required for human development and brain function. Chronic overexposure to manganese may promote potent neurotoxic effects, including disrupting mitochondrial function and induction of oxidative stress (53). However, manganese not only competes with iron for the same binding protein transferrin, but also compete with other metal ions for divalent metal transporter DMT1 which non-selectively transports multiple divalent metals (54). These findings might explain no difference observed in CSF manganese levels between two groups in our study. Chronic manganese exposure can produce cognitive deficits in rats (55), children, and young men (56), which makes the negative correlation of manganese levels with MoCA scores in young smokers easy to understand.

There is increasing evidence supporting the notion of aluminum's involvement in hastening cognitive impairment, which is thought to increase the incidence of neurological diseases, including AD (57, 58). Epidemiological studies showed that occupational exposure to aluminum was associated with poor performance on cognitive tests (59). Chronic exposure of animals to aluminum is associated with evident deficits in learning and behavioral functions. Aluminum in tobacco can be inhaled via a number of ways, such as lung and oral epithelial tissues and accumulates in the brain over time (60). Aluminum is not essential for biological activities, and if accumulated in the brain, it induces amyloid β accumulation (61). It is toxic to the nervous system and induces irreversible cognitive impairment (58). In our study, higher CSF aluminum levels with lower MoCA scores were observed in active smokers than non-smokers, further suggesting that cigarette smoking accelerates cognitive impairment.

Cigarette smoking increases lead intake. Lead alters energy metabolism and blocks the release of calcium from mitochondria leading to the formation of ROS and apoptosis of the neuron and disrupts the formation of synapses (62). Lead causes significant adverse effects on the developing brain, including cognitive and learning disabilities (63). Any fraction of lead entering the brain cannot be neglected (64). Lead crosses the blood-brain barrier (65) and preferentially accumulates in the hippocampus and cerebral cortex in mice and humans (25) with consequent cognitive deficits (66, 67). Total brain volume, the volume of gray matter in the insula and cingulum, and white matter volume in the parietal lobes were reduced in a group of workers with chronic exposure to environmental lead (68). This finding explains the decline in cognitive function caused by lead toxicity. Therefore, the higher CSF lead levels and lower MoCA scores in active smokers than non-smokers in our study suggest that cigarette smoking accelerates cognitive decline in several ways.

Additionally, in the present study, the mediation analysis has been performed with smoking habits (*X*) as the independent variable and the sample size of active smokers (*n* = 80) was not enough to calculate the mediation effect efficiently, therefore, although there were no mediation effects of metal ions observed in our results, it is estimated that cigarette smoke contains thousands of chemical compounds and toxins that are deleterious to health (69), and there are the variety of toxic heavy metals in tobacco (69), many literatures cited here have shown that cigarette smoke increases the accumulation of metal ions in tissues and fluids, that their abnormal accumulation in the nervous system could lead to heavy metal toxicity and multiple neurodegenerative diseases including cognitive impairment, and that metal ions in tobacco could enter biological tissues and organs through cigarette smoke. Moreover, our subjects are all male Han people of northern China with similar living habits and environment. Therefore, it cannot be ruled out that cigarette smoke develops into a cognitive problem through abnormal deposition of metal ions. Taking together, higher levels of CSF iron, zinc, aluminum, and lead in smokers might be associated with cigarette smoking accelerating cognitive impairment.

There are some limitations to this study. First, CSF cannot directly reflect pathological changes in neurons; nevertheless, it represents biochemical changes in the brain. Second, subjects recruited in this study had anterior cruciate ligament injuries and were not an entirely healthy population, which might be seen as a confounder when interpreting the results. Finally, if there were more subjects and female subjects, our findings might be further supported.

## Conclusion

Cigarette smoking might accelerate male cognitive impairment, as shown by lower MoCA scores and higher CSF iron, zinc, lead, and aluminum levels in active smokers. Higher levels of CSF iron, zinc, aluminum, and lead in smokers might be early evidence of cigarette smoking accelerating cognitive impairment. These broaden our understanding of cigarette smoke exposure associated with the development of neurodegenerative diseases.

## Data Availability Statement

The raw data supporting the conclusions of this article will be made available by the authors, without undue reservation.

## Ethics Statement

The studies involving human participants were reviewed and approved by Institutional Review Board of Inner Mongolian Medical University. The patients/participants provided their written informed consent to participate in this study.

## Author Contributions

FW and YL designed the study. FW, YL, CL, MW, and HL secured funding for the study. HL and QM led the drafting of the manuscript. HL led the statistical analyses. QM, XY, CL, MW, and LS collected the clinical data. All authors approved the final manuscript for submission.

## Funding

This work was supported by the following grants: The Technology Support Project of Xinjiang (2017E0267), The 10th Inner Mongolia Autonomous Region Prairie excellence Project, Natural Science Foundation of Xinjiang Province (2018D01C228 and 2018D01C239), Outstanding Youth Science and Technology Talents of Xinjiang (2017Q007), Natural Science Foundation of China (81560229 and 81760252), Beijing Natural Science Foundation (7152074), Natural Science Foundation of Inner Mongolia Autonomous Region (2020MS08191), and the Opening Project of Zhejiang Provincial Top Key Discipline of Pharmaceutical Sciences.

## Conflict of Interest

The authors declare that the research was conducted in the absence of any commercial or financial relationships that could be construed as a potential conflict of interest.

## Publisher's Note

All claims expressed in this article are solely those of the authors and do not necessarily represent those of their affiliated organizations, or those of the publisher, the editors and the reviewers. Any product that may be evaluated in this article, or claim that may be made by its manufacturer, is not guaranteed or endorsed by the publisher.

## Abbreviations

CSF: cerebral spinal fluid
AD: Alzheimer's disease
MCI: mild cognitive impairment
CNS: central nervous system
HDL: high-density lipoprotein
LDL: low-density lipoprotein
ALT: alanine aminotransferase
CHO: cholesterol
TG: triglyceride
GGT: gamma-glutamyl transferase
AST: aspartate aminotransferase
BMI: body mass index
MoCA: Montreal Cognitive Assessment
ANCOVA: Analysis of covariance
SD: standard deviation
IQR: Interquartile Range.
