# Supplementary material for: Association of Cigarette Smoking With Male Cognitive Impairment and Metal Ions in Cerebrospinal Fluid
Source: Front Psychiatry. 2021 Nov 19;12:738358. doi: 10.3389/fpsyt.2021.738358 (PMC8650691; doi:10.3389/fpsyt.2021.738358)
Supplement: Supplementary file 1 [file Table_1.PDF]

Supplement 1

The correlations between BMI/age and CSF metal levels

Variables	Non-smokers (n=100)		Active smokers (n=80)	
	BMI(r/*p*)	Age(r/*p*)	BMI(r/*p*)	Age(r/*p*)
Iron	-0.086/0.395	-0.151/0.135	0.058/0.607	-0.095/0.403
Copper	0.061/0.548	-0.030/0.763	-0.172/0.128	0.106/0.349
Zinc	-0.122/0.229	0.040/0.691	0.054/0.636	0.060/0.596
Lead	0.046/0.653	-0.067/0.509	0.096/0.396	-0.165/0.144
Aluminium	-0.013/0.896	0.072/0.476	0.040/0.725	-0.212/0.059
Manganese	-0.013/0.895	-0.085/0.403	0.037/0.746	-0.205/0.069
